# Genipin attenuates oxidative damage in periodontal tissues by alleviating mitochondrial dysfunction and abnormal glucose uptake through inhibition of UCP2

**DOI:** 10.3389/fphar.2025.1446574

**Published:** 2025-03-26

**Authors:** Yu Deng, Ruobing Fu, Yaqi Shang, Mengting Hu, Xirui Xin, Yubo Hou, Weixian Yu, Xinchan Liu

**Affiliations:** ^1^ Department of Periodontology, Hospital of Stomatology, Jilin University, Changchun, China; ^2^ Department of Periodontics, School and Hospital of Stomatology, Wenzhou Medical University, Wenzhou, China; ^3^ Department of Geriatric Stomatology, Hospital of Stomatology, Jilin University, Changchun, China; ^4^ Jilin Provincial Key Laboratory of Tooth Development and Bone Remodeling, Hospital of Stomatology, Jilin University, Changchun, China; ^5^ VIP General Department, Hospital of Stomatology, Jilin University, Changchun, China

**Keywords:** genipin, periodontitis, UCP2, mitochondrial dysfunction, glucose uptake

## Abstract

**Introduction:**

Periodontitis is a chronic inflammatory disease closely associated with mitochondrial dysfunction. Uncoupling protein 2 (UCP2), located in the inner membrane of mitochondria, reduces mitochondrial membrane potential (MMP) and adenosine triphosphate (ATP) synthesis by promoting proton leakage across the membrane. This leads to decreased energy metabolism efficiency, impairing cellular glucose uptake, and disrupting intracellular energy balance. Genipin (GP), a recognized UCP2 inhibitor, exhibits anti-inflammatory and antioxidant properties. This study aimed to investigate the specific role of GP in periodontal tissue redox signaling and the potential mechanism of UCP2 in the development of periodontitis.

**Methods:**

In this study, we constructed a model of H_2_O_2_-induced oxidative stress in human periodontal ligament cells (hPDLCs). *In vivo*, a rat periodontitis model was established to evaluate the effects and mechanisms of GP in alleviating oxidative damage in periodontal tissues and cells.

**Results:**

Cell experiments showed that GP effectively alleviated H_2_O_2_-induced mitochondrial dysfunction and oxidative damage in hPDLCs by inhibiting UCP2 expression and function, restoring cell viability, and reducing cell apoptosis. Additionally, GP intervention increased the expression of glucose transporter 4 (GLUT4), thereby promoting cellular glucose uptake. The results of animal experiments demonstrated that GP intervention reduced alveolar bone resorption and periodontal tissue destruction in rats with periodontitis, inhibited osteoclast differentiation, improved mitochondrial dysfunction in periodontal tissue, promoted GLUT4 expression, and reduced oxidative stress levels and cell apoptosis.

**Discussion:**

GP regulates oxidative damage in periodontal tissues by maintaining mitochondrial homeostasis, promoting glucose transporter expression, and enhancing glucose uptake, with UCP2 playing a central role.

## 1 Introduction

Periodontitis is an ongoing and escalating inflammatory state defined by the pathological breakdown of periodontal tissues. It eventually leads to tooth loss, while affecting chewing and aesthetics (Li et al., 2023). Periodontitis is widespread, with approximately 11 percent of the world’s population affected by severe periodontitis (Eke et al., 2018). Mounting evidence suggests that oxidative stress centrally contributes to the deterioration of tissues linked with periodontitis (Wang et al., 2017; Chen et al., 2019b; Sczepanik et al., 2020). Oxidative stress leads to periodontitis by directly damaging connective tissue and promoting osteoclastogenesis (Baltacıoğlu et al., 2014). Moreover, individuals afflicted with periodontitis exhibit elevated levels of markers indicating oxidative stress (Chen et al., 2019b). Consequently, additional investigation into mechanisms linked to oxidative stress is imperative to enhance the development of successful treatments for periodontitis.

Mitochondria as the primary sources of reactive oxygen species (ROS), are frequently targeted by ROS, resulting in structural alterations and dysfunction. Mitochondrial dysfunction is commonly linked to various diseases, primarily characterized by reduced adenosine triphosphate (ATP) production, reduced oxidative phosphorylation efficiency, and increased oxidative stress (Hoogstraten et al., 2024; Zong et al., 2024). Research demonstrates a strong correlation between mitochondrial dysfunction and the initiation as well as advancement of periodontitis (Chen et al., 2019c; Cao et al., 2023). Uncoupling proteins (UCPs), a set of proteins located in the inner mitochondrial membrane, influence mitochondrial function and energy metabolism by uncoupling oxidative phosphorylation (Barnstable et al., 2022). Among these proteins, mitochondrial uncoupling protein 2 (UCP2) is the most prevalent. UCP2 regulates mitochondrial membrane potential (MMP), ATP levels, and ROS production (Hirschenson et al., 2022). By promoting proton leakage across membranes, UCP2 reduces MMP and ATP synthesis, leading to decreased energy metabolism efficiency, which may impair cellular glucose utilization (Toda et al., 2016).

Glucose is a vital fuel and essential metabolic substrate, and its uptake and metabolism ensure that cells have access to energy. Due to its hydrophilia, glucose cannot directly permeate cell membranes. Glucose uptake is enabled by members of the glucose transporter protein family, which includes 14 identified members. Among these, glucose transporter proteins 1, 2, 3, and 4 (GLUT1, GLUT2, GLUT3, and GLUT4) exhibit a high affinity for glucose, and play significant roles in facilitating glucose uptake (Kato et al., 2004; Bogan, 2022). Studies indicate that mitochondrial dysfunction can disrupt intracellular signaling pathways, potentially reducing the expression or activity of glucose transporters, thereby decreasing glucose uptake efficiency and impacting glucose metabolism, which in turn affects the intracellular energy balance (Carvalho and Moreira, 2023; Fang et al., 2023; Kong et al., 2024). Numerous studies have demonstrated a notable correlation between compromised glucose metabolism and both periodontal bleeding and the development of severe periodontitis (Pérez et al., 2017). Moreover, enhancing cellular glucose uptake can improve gum wound healing (Li et al., 2022a). However, the potential effects of decreased cellular glucose uptake on periodontitis remain to be elucidated.

Genipin (GP) is an aglycone originating from iridoid glycosides, primarily found in the fruits of the Gardenia rubiaceae plant family, which exhibits a wide array of biological and pharmacological functions, notably featuring potent antioxidant and anti-inflammatory properties. Its historical use in traditional oriental medicine spans various inflammation-related conditions (Zhang et al., 2021; Cho, 2022). Crucially, GP serves as a conventional pharmacological inhibitor of UCP2, widely utilized in studies related to UCP2 (Cho, 2022).

In this study, we constructed an H_2_O_2_-induced oxidative stress model *in vitro* and a rat periodontitis model to investigate the potential protective effects of GP against periodontitis in rats, the associated molecular mechanisms involved, and the potential role of UCP2 in this process.

## 2 Materials and methods

### 2.1 Cell isolation and culture

Patient-derived hPDLCs can better reflect the characteristics of periodontal tissue and more accurately simulate the disease process and cell behavior. Especially in studies of periodontal tissue repair, periodontal inflammatory responses, and other processes, patient-derived cells have greater clinical translation potential. Shi et al.’s studies all extracted hPDLCs from patients (Shi et al., 2021; Mei et al., 2024). This study was approved by the Medical Ethics Committee of the Hospital of Stomatology, Jilin University (No. 202232). All patients have signed an informed consent form. The middle root surface tissue of healthy third molars (with an average age of 19) were scraped and cut into 1 mm^3^ tissue blocks. The culture of hPDLCs proceeded as previously reported (Shi et al., 2021). The cells were passaged when they were between 70 and 80 percent confluent. Four to eight passages of cells were obtained for the experiment.

### 2.2 Experimental model and treatment conditions

To select the optimal concentrations of H_2_O_2_ (Sigma, United States) and the drug toxicity range of GP (Aladdin, China). GP dissolved in DMSO.96-well plates, with 5 × 10^3^ cells per well, were utilized to cultivate hPDLCs at various concentrations of H_2_O_2_ or GP. The Cell Counting Kit-8 (CCK-8) (Beyotime, China) used the kit’s instructions to determine the viability of the cells. The microplate assay was used to determine OD values at 450 nm. All data were obtained from three independent experiments.

### 2.3 MMP detection

The MMP of hPDLCs was determined using JC-1 mitochondrial membrane potential detection kit (Solarbio Science, China). JC-1 working solution was prepared according to the instructions, and hPDLCs were cultured with working solution at 37°C away from light for 20 min. The nucleus were stained with Hoechst (Beyotime, China). The hPDLCs was observed under a fluorescence microscope (Olympus, DP74).

### 2.4 Immunofluorescence

After paraffin sections were dewaxed and rehydrated, antigen retrieval was performed. The tissues were then incubated with primary antibodies against UCP2 (1:50; Affinity, United States) overnight at 4°C. Subsequently, the samples were incubated with a secondary antibody at a dilution of 1:1,000 and followed by Hoechst staining. Fluorescence microscopy (Olympus, Japan) was used to observe and quantify positive cells (Yue et al., 2021).

### 2.5 Glucose uptake assay

hPDLCs were incubated overnight in serum-free basal medium. After PBS washes, the cells underwent pretreatment with GP, followed by H_2_O_2_ treatment. Glucose uptake was evaluated after a 60 min incubation with 100 μM 2-NBDG (2-[N-(7-Nitrobenz-2-oxa-1,3-diazol-4-yl) Amino]-2-Deoxyglucose, 2-NBDG) (MCE, United States). The cells were rinsed three times with PBS to eliminate unabsorbed 2-NBDG, and the fluorescence of the cells was measured at 540/570 nm.

### 2.6 Animals

Twenty-four 8-week-old male Wistar rats, weighing about 200–220 g, were obtained from the Laboratory Animal Center of Jilin University. They were fed in a 12-h daily cycle at 22°C–25°C and supplemented with free food and water during the week before the surgeries and therapies. All experiments involving animals were conducted in strict adherence to the Guide for Ethical Review of Laboratory Animals - Animal Welfare and were approved by the Animal Ethics Committee of Jilin University (NO. SY202207103).

### 2.7 Animal models and group allocation

Twenty-four rats were randomly divided into three groups: control (C), experimental periodontitis (P), and experimental periodontitis treated with GP (P+GP). In accordance with our prior research (Ding et al., 2022; Li et al., 2022b), the rats in the P and P+GP groups were intraperitoneally sedated with 2% pentobarbital sodium (0.2 mL/100 g) before a diameter of 0.2 mm orthodontic wire was layed in the neck of the maxillary first molar. The ligatures were maintained for 2 weeks with daily checks. If the ligature is removed, a new ligature will be added. GP was dissolved in normal saline. During this period (Xu et al., 2020; Zhang et al., 2021), the rats in the P+GP group were given GP (50 mg/kg/day) by gavage. The C and P groups received the same dosage of normal saline. The method of GP administration was similar to previous studies. All the rats were euthanized after 2 weeks. The upper jaw bone was then gathered for the subsequent examinations.

### 2.8 Alveolar bone of microarchitecture analysis and hematoxylin and eosin (H&E) staining

The left maxilla had been scanned using a micro-CT (Μct50, Scanco). The parameters for the micro-CT: 200 Ma, 300 ms of exposure duration, and 70 Kv (a voxel size of 10 μm) (Li et al., 2022b). Analysis of relevant bone biological indicators: bone mineral density (BMD), bone volume/total volume (BV/TV) and trabecular thickness (Tb. Th). The distance and mean value between the cement-enamel junction (CEJ) and alveolar bone crest (ABC) were measured. The samples remove wax from sections, and H&E staining was performed.

### 2.9 Immunohistochemical analysis

Immunohistochemistry was performed to examine UCP2 and GLUT4 levels in periodontal tissues. The techniques and protocols for immunohistochemistry were executed following the methodologies previously described in our reports (Li et al., 2022b). Information on primary antibodies is given below: UCP2 (1:50; Affinity, United States) and GLUT4 (1:50; Proteintech, United States).

### 2.10 Mitochondrial ROS levels

The MitoSOX Red reagent was used to quantify mtROS (Thermo Scientific, United States) (Sun et al., 2017; Chen et al., 2021). The cells and frozen periodontal tissue sections (4 μm thick) were incubated with a 5 μM MitoSox™ working solution. Hoechst was used to color the nuclei. The findings were obtained using fluorescence microscopy (Olympus, DP74), and ImageJ was utilized to assess findings.

### 2.11 ATP synthesis assays

The hPDLCs or periodontal tissue samples were lysed and the supernatant was obtained after centrifugation. Following the manufacturer’s instructions, an enhanced ATP Assay Kit (Solarbio Science, China) was employed to evaluate the ATP concentration.

### 2.12 Tartrate-resistant acidic phosphatase (TRAP) staining

The osteoclasts were counted with the TRAP staining kit (Sigma, United States). Remove wax from slices, heat distilled water to 37°C for the configuration of TRAP working fluid, incubate at 37°C for 1 h away from light, rinse with running water. Finally, methyl green was re-stained for 5 min.

### 2.13 Superoxide dismutase (SOD) and malondialdehyde (MDA) activity assay

After treatment, the cells in each group were washed with PBS, lysed with a lysis buffer, and the supernatant was collected by centrifugation. Subsequent procedures were performed following the SOD and MDA assay kit instructions (Jiancheng Bioengineering Institute, China). The levels of SOD and MDA in rat serum were also measured according to the kit instructions.

### 2.14 Western blot analysis

hPDLCs and periodontal tissues were treated with RIPA lysate plus PMSF (the ratio of RIPA to PMSF was 100:1). Subsequently, the protein concentration was quantified using the BCA protein assay kit. The sample is loaded and electrophoresis, and then transferred to a polyvinylidene fluoride (PVDF) membrane. After 1 h of sealing with 5% skim milk at room temperature, The membrane was exposed overnight to primary antibodies against Bax (1:2,000; Proteintech, United States), Bcl2 (1:1,000; Proteintech, United States), Casepase-3 (1:1,000; Cell Signaling Technology, United States), UCP2 (1:1,000; Affinity, United States), GLUT4 (1:1,000; Proteintech, United States), β-actin (1:8,000; Proteintech, United States), and GAPDH (1:8,000; Proteintech, United States). Next, after three rinses, the membrane was incubated with secondary antibodies and then imaged with BeyoECL Moon. The grayscale value of band intensity was measured by ImageJ software.

### 2.15 Statistical analysis

All data are reported as the mean ± SD of at least three independent experiments. Differences between two groups were analyzed using the unpaired t-test for parametric data. Three or more groups were compared using one-way analysis of variance (ANOVA).Statistical analysis was performed using GraphPad Prism 10.1.2. Statistical significance was set at *p* < 0.05.

## 3 Results

### 3.1 Oxidative stress-induced UCP2 overexpression mediates cellular injury and apoptosis

**FIGURE 1:**
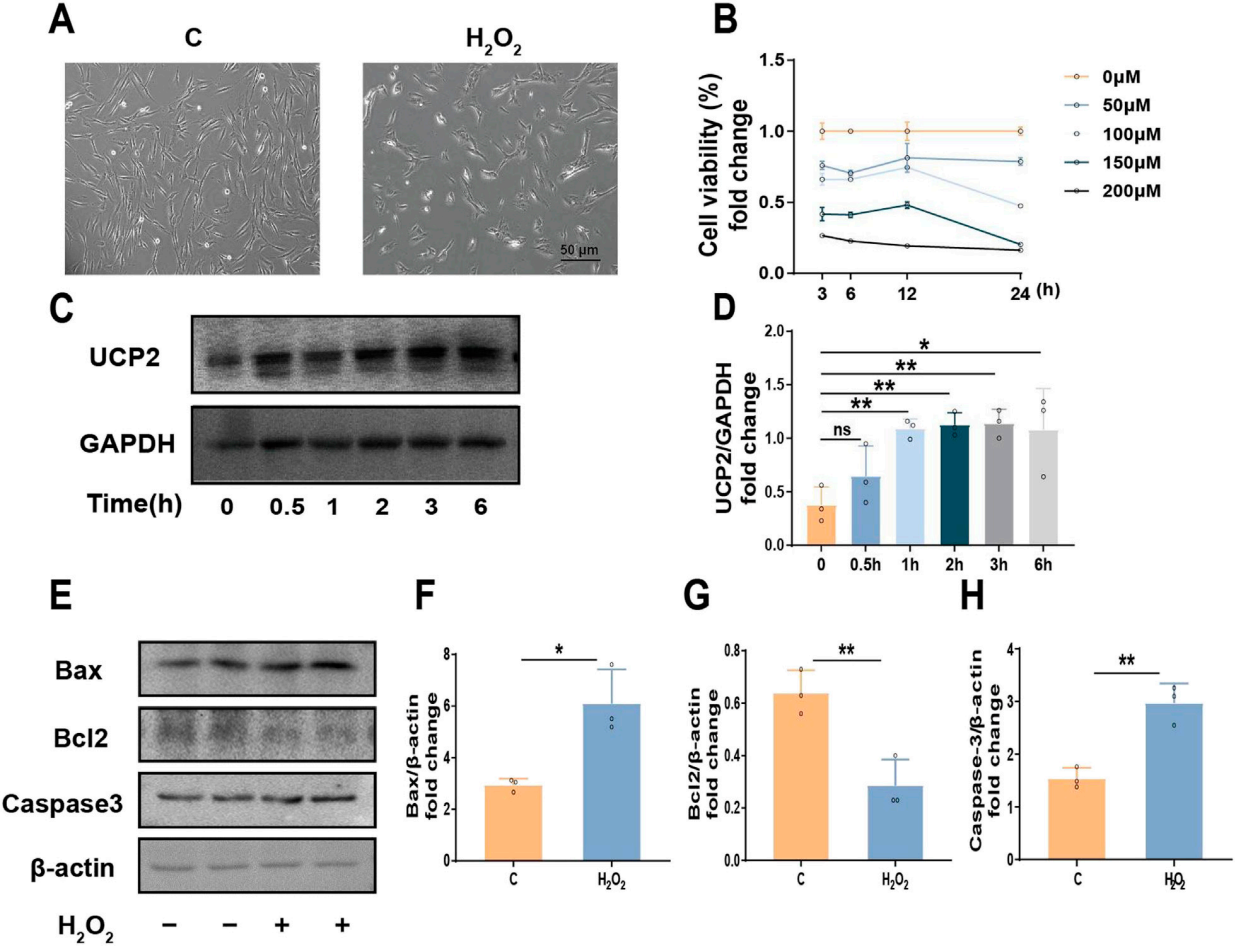
UCP2 could potentially play a role in inducing apoptosis in hPDLCs. **(A)** Morphology of hPDLCs under H_2_O_2_ (scale = 50 μm). **(B)** CCK-8 test to assess cell viability in amount. **(C, D)** Western blot detected UCP2 expression levels at different times under 100 μM H_2_O_2_ and quantitative analysis. **(E–H)** The protein levels detected by Western blot of Bax, Bcl2 and Caspase-3 in hPDLCs and quantitative analysis. All data were based on three independent experiments and presented as the mean ± SD. ns, not significant; *p < 0.05; **p < 0.01.

In order to replicate the oxidative stress microenvironment typical of periodontitis, hPDLCs were subjected to diverse concentrations of H_2_O_2_, and cellular activity was evaluated. The findings indicated that cell viability decreased in response to H_2_O_2_ treatment in a dose- and time-dependent fashion. Notably 100 μM was the half-inhibitory dose (Figures 1A, B). Therefore, we identified 100 μM H_2_O_2_ as the most suitable concentration for subsequent experiments. In addition, we observed that UCP2 expression increased with time under the action of H_2_O_2_, reaching a peak at 3 h (Figures 1C, D). when cells were exposed to 100 μM H_2_O_2_, the expression of Bax and Caspase3 were upregulated, while the expression of Bcl2 was downregulated (Figures 1E–H). In summary, we suggested that UCP2 might participate in H_2_O_2_-induced hPDLCs damage and mediate apoptosis.

### 3.2 GP effectively alleviates H_2_O_2_-induced hPDLCs injury and apoptosis by inhibiting UCP2

**FIGURE 2:**
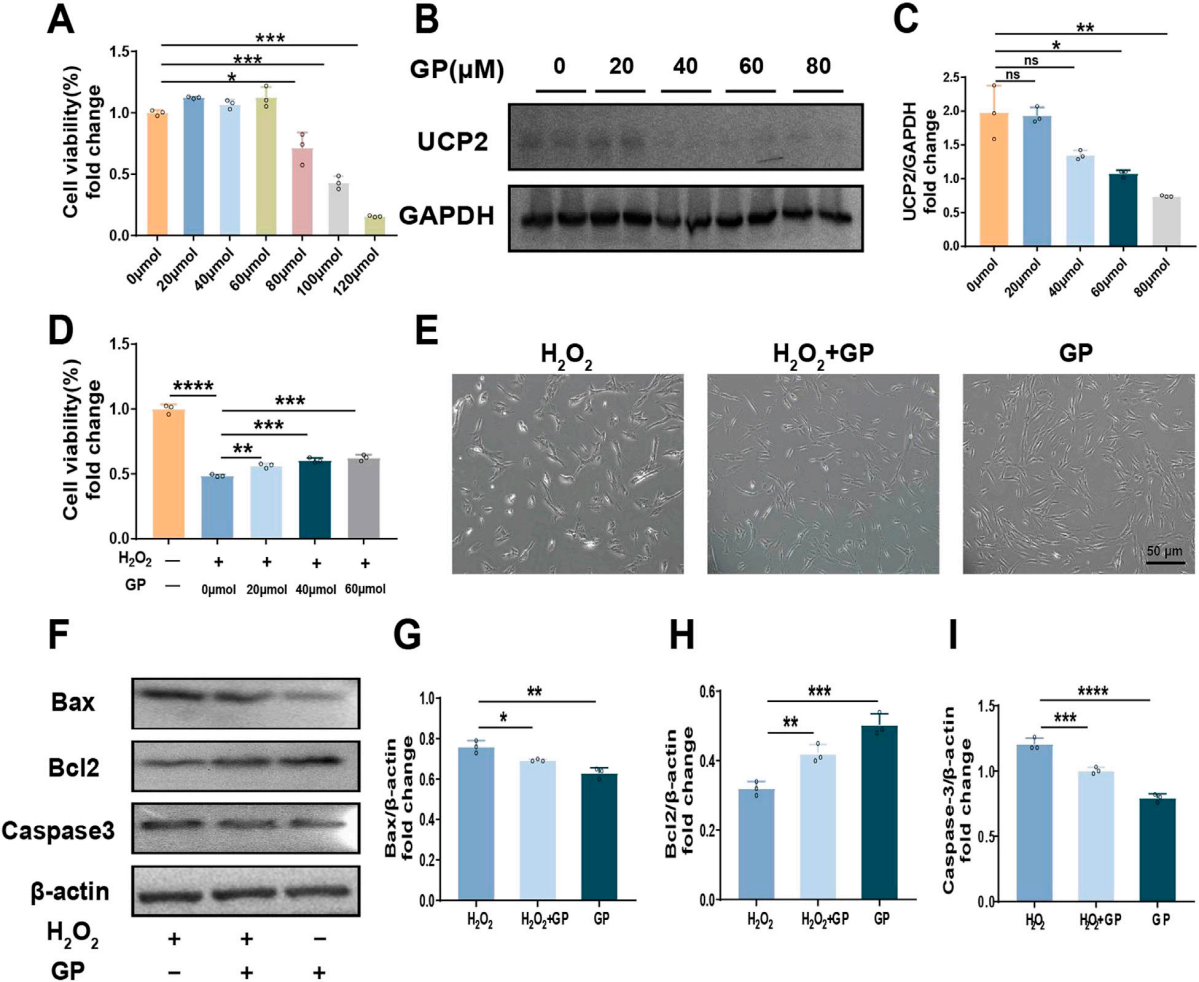
Inhibition of UCP2 can reduce H_2_O_2_-induced apoptosis of hPDLCs. **(A)** CCK-8 test to evaluate the quantity of viable cells. **(B, C)** Western blot detected UCP2 expression levels at different concentration of GP and quantitative analysis. **(D)** Proliferation of hPDLCs in 100 μM H_2_O_2_ with various GP concentrations used as pretreatment. **(E)** Morphology of hPDLCs after GP treatment (scale = 50 μm). **(F–I)** Expression of Bax, Bcl2 and Caspase-3 in hPDLCs and quantitative analysis. All data were based on three independent experiments and presented as the mean ± SD. ns, not significant; *p < 0.05; **p < 0.01; ***p < 0.001; ****p < 0.0001.

Different doses (0–120 µM) of GP were treated with hPDLCs for 24 h to detect cytotoxicity. The results showed that GP exceeding 60 µM produced cytotoxicity compared to group C, so 60 μM GP was selected for subsequent experiments (Figure 2A). In addition, we observed that UCP2 expression decreased with increasing concentration of GP (Figures 2B, C). After pretreating hPDLCs with GP (60 μM, 30 min), we observed an enhancement in cell viability compared to the H_2_O_2_ group, suggesting that GP shows promise in reinstating cell viability and fostering cell proliferation to a certain degree (Figures 2D, E). It is worth noting that after GP intervention, the expression of Bax and Caspase3 in cells decreased, while that of Bcl2 increased (Figures 2F–I). In conclusion, GP can effectively reduce H_2_O_2_-induced apoptosis in hPDLCs by inhibiting UCP2 expression.

### 3.3 GP alleviates H_2_O_2_-induced hPDLCs mitochondrial dysfunction and decreased glucose uptake via UCP2

**FIGURE 3:**
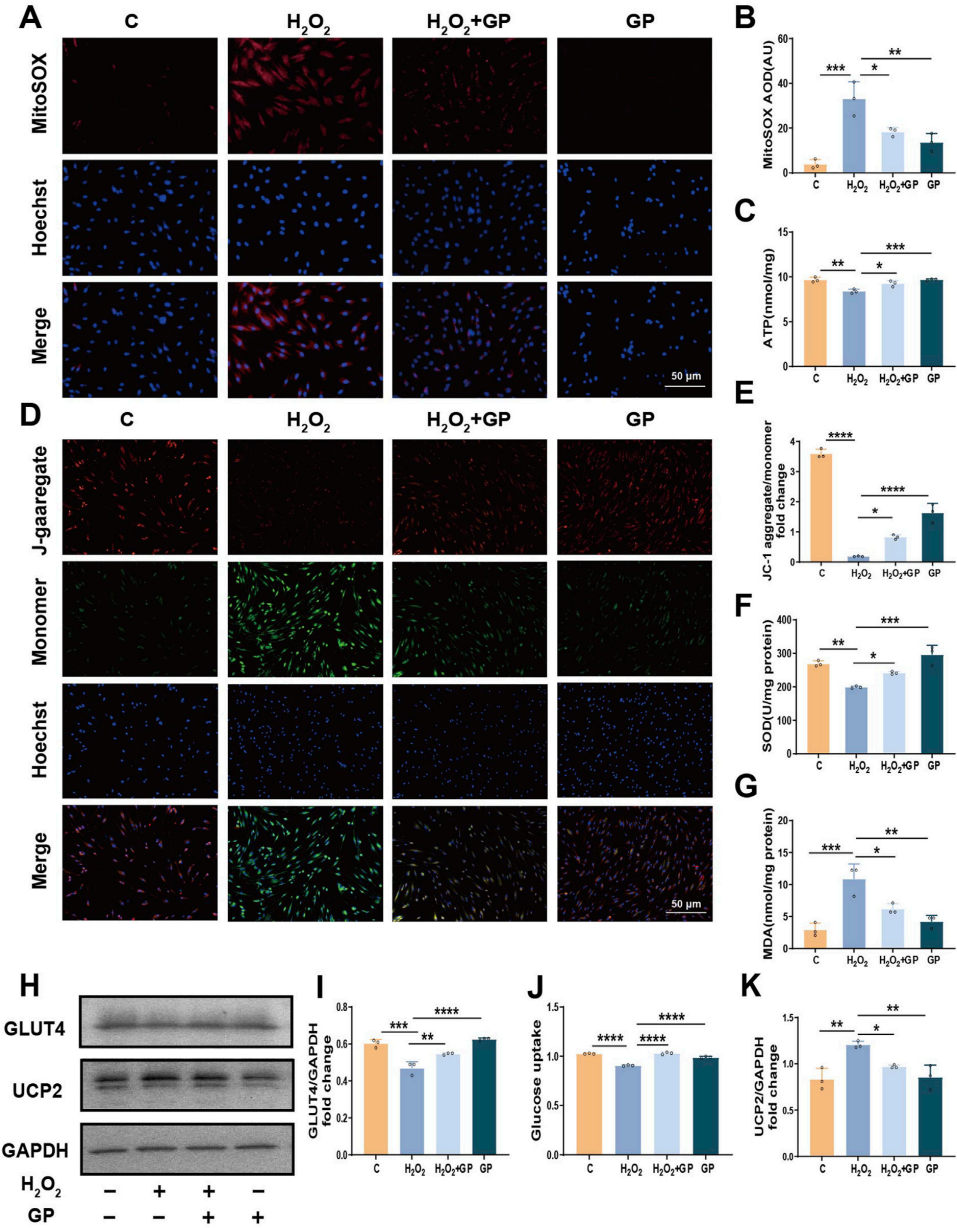
GP inhibition of UCP2 alleviates mitochondrial dysfunction and glucose uptake defects in hPDLCs. **(A, B)** MitoSOX representative images (scale = 50 μm) and intensity unit quantification. **(C)** Determination of ATP levels in hPDLCs. **(D,E)** JC-1 images (scale = 50 μm) and intensity unit quantification. **(F, G)** SOD and MDA activity in hPDLCs. **(H)** The protein levels of UCP2 and GLUT4 in hPDLCs. **(I)** GLUT4 level relative to GAPDH. **(J)** Changes in glucose uptake in hPDLCs. **(K)** UCP2 level relative to GAPDH. All data were based on three independent experiments and presented as the mean ± SD. *p < 0.05; **p < 0.01; ***p < 0.001; ****p < 0.0001.

First, MitoSOX was used to detect mtROS. H_2_O_2_ stimulation significantly increased the amount of mtROS, and the level of mtROS decreased after GP intervention (Figures 3A, B). As a marker of metabolic activity, the ATP level in the H_2_O_2_+GP group showed an elevation compared to the H_2_O_2_ group (Figure 3C). MMP was assessed using JC-1 staining. In hPDLCs subjected to H_2_O_2_ exposure, there was a gradual accumulation of green fluorescent monomers, accompanied by a weakening of red fluorescent aggregates. Subsequent quantitative analysis revealed that the ratio of red to green fluorescence intensity in the H_2_O_2_+GP group surpassed that of the H_2_O_2_ group (Figures 3D, E). H_2_O_2_ markedly induces intracellular ROS generation. Following exposure to H_2_O_2_, the expression of SOD, a primary antioxidant enzyme, declined. Meanwhile, MDA, a crucial marker of membrane lipid peroxidation, exhibited a significant increase, which was reversed by GP intervention (Figures 3F,G). H_2_O_2_-induced impaired glucose uptake in cells can be reflected in decreased expression of glucose transporter and decreased glucose uptake. We detected the expression of GLUT4 protein in hPDLCs, stimulation with H_2_O_2_ notably reduced GLUT4 expression, whereas GP intervention substantially augmented GLUT4 levels in hPDLCs (Figures 3H, I). In order to further examine GP’s regulation of glucose uptake in cells, we measured glucose uptake with 2-NBDG and found that GP mitigated the H_2_O_2_-induced reduction in cellular glucose uptake (Figure 3J). Meanwhile as an inhibitor of UCP2, the expression of UCP2 protein decreased after GP intervention (Figures 3H,K). These results suggest that GP may have alleviated mitochondrial dysfunction, suppressed oxidative damage in hPDLCs, and increased GLUT4 expression and GLUT4-mediated glucose uptake by inhibiting UCP2.

### 3.4 GP mitigate tissue damage in a rat model of ligation-induced periodontitis

**FIGURE 4:**
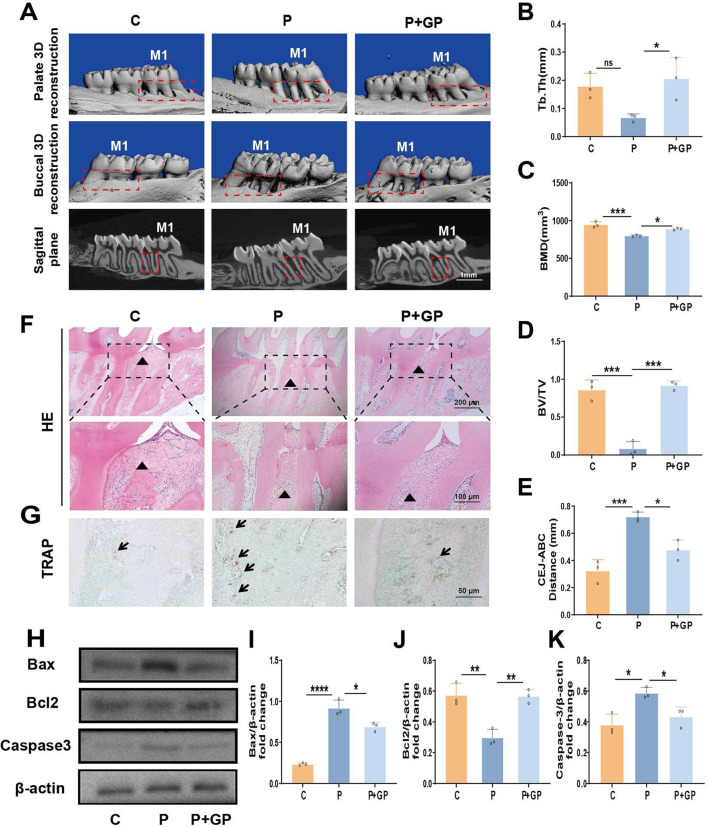
Impact of GP on rat periodontitis. **(A)** Micro-CT scan results (scale = 1 mm). M1 represents the maxillary first molar, and the red frame represents the alveolar bone. **(B)** Tb.Th. **(C)** BMD. **(D)** BV/TV. **(E)** Distance from CEJ to ABC. **(F)** H&E staining results (magnification ×40, ×100), alveolar ridge represented by black triangle. **(G)** TRAP dyeing result. The black arrow indicates osteoclasts (scale = 20 μm). **(H–K)** The protein levels of Bax, Bcl2 and Caspase-3 in periodontal tissue of rats. Data shown are mean ± SD (n ≥ 3). *p < 0.05; **p < 0.01; ***p < 0.001; ****p < 0.0001.

We successfully established a ligation induced periodontitis model in rats. Micro-CT indicated a decrease in Tb.Th, BMD and BV/TV in the root fork area of the maxillary first molar in rats of group P, along with an increase in the distance from the CEJ to the ABC compared to group C. These observations suggest a substantial degree of alveolar bone resorption in rats of group P (Figures 4A–E). Furthermore, H&E staining of periodontal tissue from rats in group P revealed the dissolution of collagen fibers in the connective tissue, pronounced loss of attachment, and substantial infiltration of inflammatory cells (Figure 4F). TRAP staining revealed a notable elevation in the count of osteoclasts in rats of group P, providing supplementary evidence for increased bone resorption in the alveolar bone (Figure 4G). Nevertheless, the observed pathological symptoms showed improvement in the P+GP group. Additionally, there was a decrease in the expression of apoptosis-related factors in periodontal tissues after GP treatment. (Figures 4H–K). The results above indicate that GP has the potential to effectively mitigate periodontal tissue damage in rats by diminishing the level of apoptosis.

### 3.5 GP mitigates oxidative damage in rat periodontal tissues through the inhibition of UCP2

**FIGURE 5:**
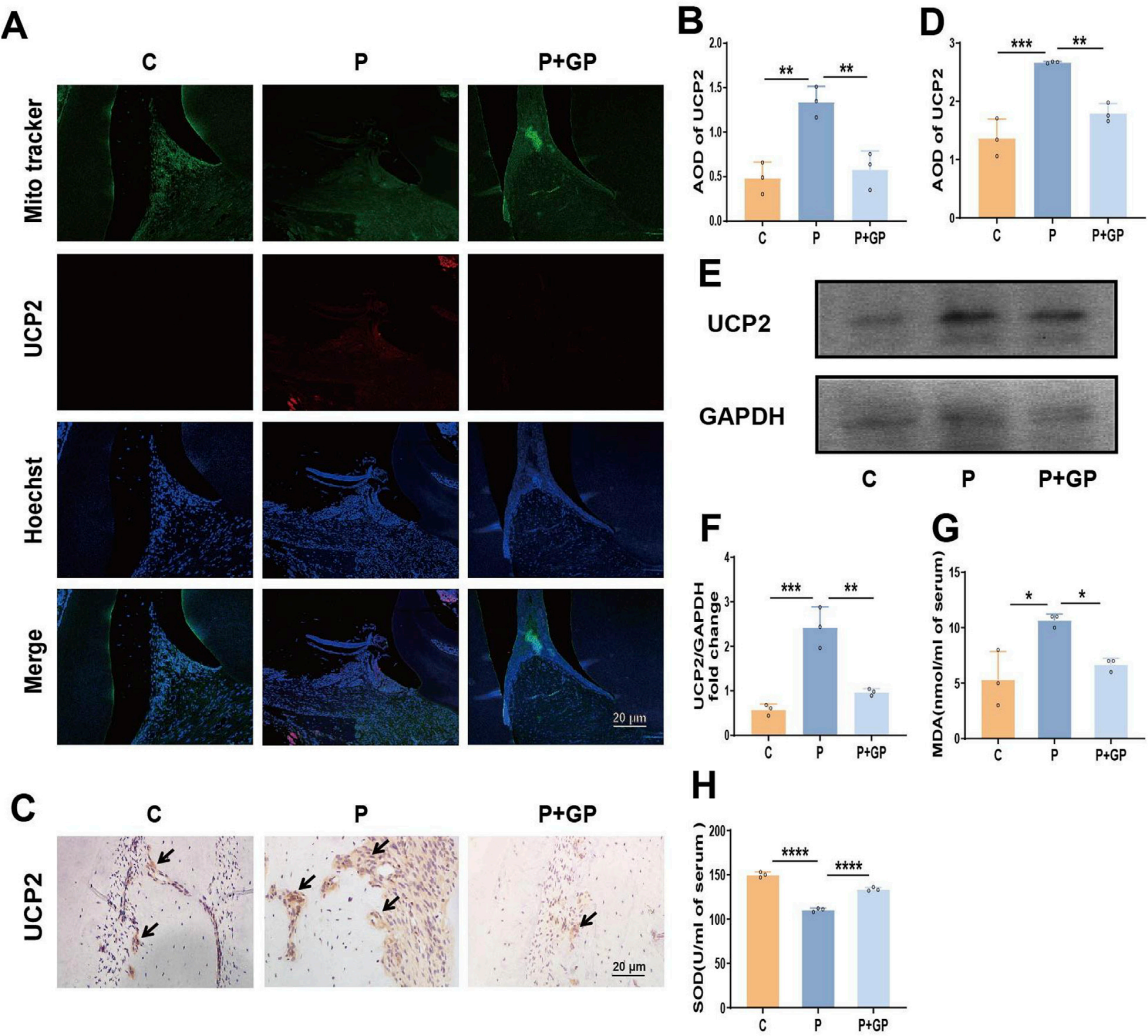
GP mitigates oxidative damage in rat periodontal tissues by suppressing UCP2 activity. **(A, B)** Fluorescence microscope image and quantitative analysis of UCP2 in periodontal tissue of rats (Scale bar = 20 μm). **(C, D)** Immunohistochemical staining of UCP2 in rats’ periodontal tissue and the average optical density (Scale bar = 20 μm). Black arrows represent the positive expression of UCP2 protein. **(E, F)** The protein level detected by Western blot of UCP2 and the level relative to GAPDH. **(G, H)** MDA and SOD activity in rat serum. Data shown are mean ± SD (n = 3). *p < 0.05; **p < 0.01; ***p < 0.00; ****p < 0.0001.

Given the significant role of UCP2 in H_2_O_2_-induced mitochondrial dysfunction and reduced glucose uptake in hPDLCs, we further tested the role of UCP2 in periodontitis. Firstly immunofluorescence results showed that the mitochondria-targeting dye Mito-tracker green overlapped with the red fluorescence of UCP2, indicating that UCP2 was expressed in mitochondria. Meanwhile, UCP2 expression was enhanced in the periodontal tissues of rats in group P, whereas UCP2 expression was significantly reduced after GP intervention (Figures 5A, B). Immunohistochemical staining and Western blot also showed that UCP2 expression in the periodontal tissue of rats in group P was upregulated compared with that in group C, while UCP2 expression in group P+GP was lower than that in group P (Figures 5C–F). Levels of MDA and SOD, markers of oxidative stress, were assessed in rat serum. In the group receiving GP intervention, MDA levels decreased while SOD levels increased compared to the P group (Figures 5G, H).

### 3.6 Effects of GP on mitochondrial function and GLUT4 in periodontal tissue of rats

**FIGURE 6:**
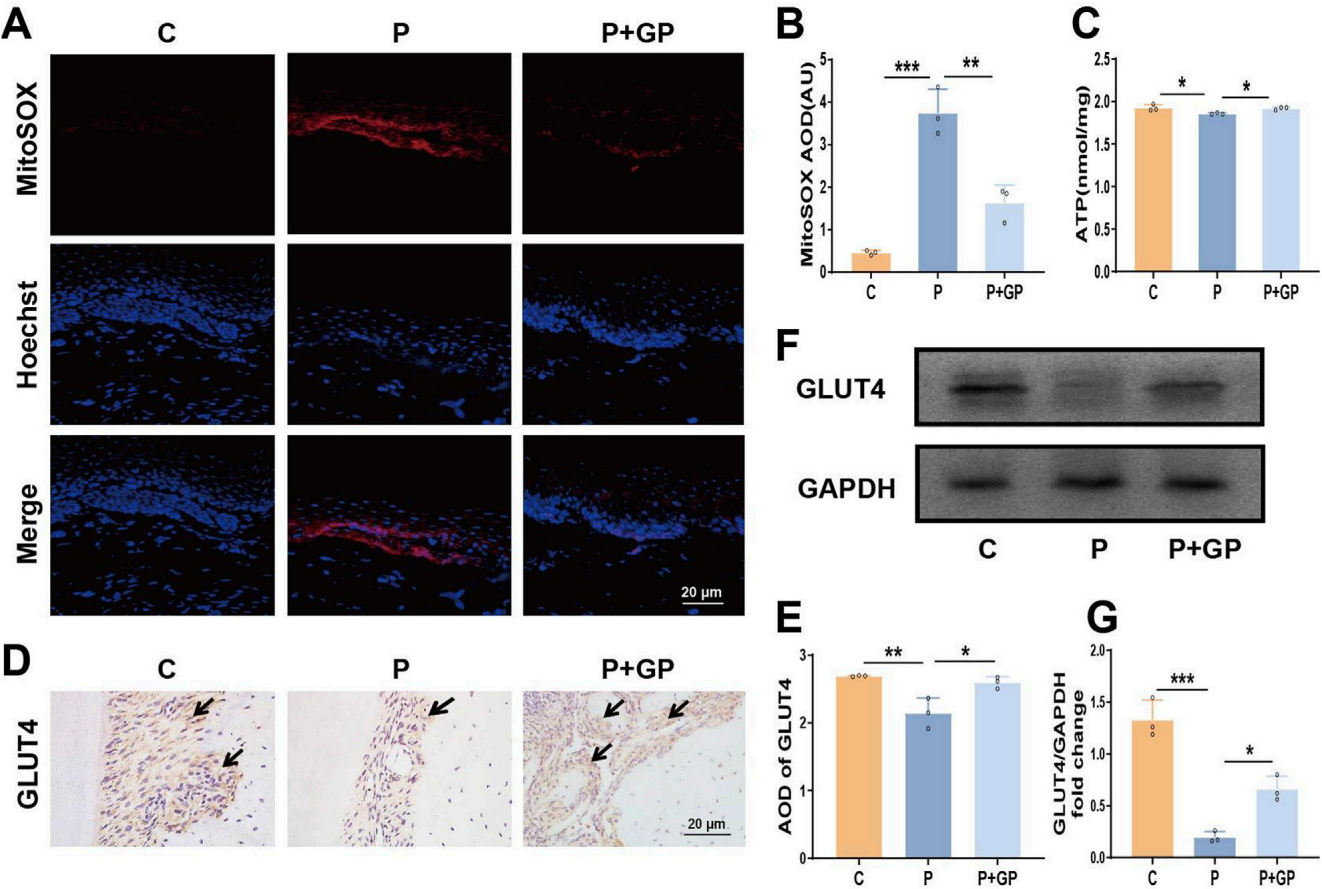
GP alleviated mitochondrial dysfunction and increased GLUT4 expression in periodontal tissue of rats. **(A, B)** Images of MitoSOX and quantification of intensity units (Scale bar = 20 μm). **(C)** Determination of ATP levels in different group. **(D, E)** Immunohistochemical staining of GLUT4 in rats’ periodontal tissue and the average optical density (Scale bar = 20 μm). Black arrows represent the positive expression of GLUT4 protein. **(F, G)** The protein level detected by Western blot of GLUT4 in periodontal tissue of rats and the level relative to GAPDH. Data shown are mean ± SD (n = 3). *p < 0.05; **p < 0.01; ***p < 0.001.

MitoSOX staining was employed to identify mtROS, revealing a significant elevation in mtROS expression within the periodontal tissues of rats in group P (Figures 6A, B). In addition, ATP synthesis in periodontal tissues of rats in group P was significantly reduced (Figure 6C), while the above indicators were reversed after GP intervention. Immunohistochemical staining and Western blot analysis were used to evaluate the expression of GLUT4 protein in periodontal tissues. Compared with group C, the expression of GLUT4 in periodontal tissue of group P was significantly decreased, and compared with group P, the expression of GLUT4 was increased after GP intervention (Figures 6D–G). The results showed that GP alleviated mitochondrial dysfunction and increased GLUT4 expression in periodontal tissue of rats.

## 4 Discussion

Presently, scholarly consensus acknowledges that adjusting the redox balance within periodontal tissue can effectively alleviate the symptoms of periodontitis. Nonetheless, effective interventions for treating periodontitis remain scarce. Our research indicates that GP mitigates periodontitis in rat models. The potential mechanisms involve safeguarding mitochondrial function and enhancing glucose uptake, with UCP2 playing a pivotal role in this process. Our results additionally offer compelling support for preserving mitochondrial function and enhancing glucose uptake, presenting a novel avenue for preventing periodontitis.

Mitochondria play a crucial role in cellular function. Mitochondrial dysfunction can activate intracellular signaling pathways that lead to apoptosis, a process involved in the progression of nearly all diseases (Chen et al., 2019a). Relevant literature indicates, characteristic signs of mitochondrial dysfunction encompass alterations in mitochondrial structure and MMP, accumulation of mtROS, and a decrease in ATP levels (Chen et al., 2019c; Sun et al., 2023). In this study, we observed that excess ROS disrupts mitochondrial redox homeostasis and leads to mitochondrial dysfunction. The results of *in vitro* experiments showed that H_2_O_2_ inhibited ATP production of hPDLCs, decreased MMP, increased mtROS production, increased oxidative stress level and UCP2 expression. Similarly, in the periodontitis group, ATP production decreased, mtROS production increased, oxidative stress levels rose, and UCP2 expression was elevated. However, after inhibition of UCP2 by GP, these effects were reversed, and mitochondrial dysfunction was reduced. Studies have shown that mitochondrial dysfunction can activate mitochondria-dependent apoptosis (Bock and Tait, 2020). Aligned with this discovery, our study demonstrated a notable increase in apoptosis levels in hPDLCs treated with H_2_O_2_. Furthermore, an increase in apoptosis was observed in the periodontal tissue of rats with periodontitis, accompanied by deepening of the periodontal pocket, heightened attachment loss, increased osteoclast numbers, and a marked decrease in alveolar bone height. After GP intervention, apoptosis levels in cells and tissues were reduced, and periodontal tissue destruction was alleviated. These findings confirmed that when mitochondrial dysfunction occurs, UCP2 expression increase, which will promote the occurrence of apoptosis, and that GP inhibition of UCP2 can mitigate mitochondrial dysfunction and apoptosis, thereby alleviating periodontitis progression.

UCP2 functions as a mitochondrial proton anion regulator, governing the generation of mitochondrial superoxide anions (Tian et al., 2018). Studies have found that UCP2 acts as an antioxidant in some cases and mediates pro-oxidation in others. However, the full comprehension of UCP2’s involvement in the advancement of periodontitis remains incomplete. The traditional antioxidative effect of UCP2 is attributed to its capacity to increase proton leakage and dissipate MMP, consequently diminishing reverse electron transfer (Arsenijevic et al., 2000). In our study, we observed increased UCP2 expression in the periodontal tissues of periodontitis rats and H_2_O_2_-treated hPDLCs, and UCP2 dissipates MMP under oxidative stress. However, when UCP2 expression was increased, mtROS was increased, while when UCP2 was inhibited by GP, mtROS production was significantly reduced and ATP and MMP levels increased. Rangarajan S et al. found that persistently elevated UCP2 levels correlate with heightened ROS production, modified redox status and cellular bioenergy, compromised fatty acid oxidation, and the initiation of cellular senescence (Rangarajan et al., 2022). At the same time, studies have shown that overexpression of UCP2 mediates excessive uncoupling of mitochondrial oxidative phosphorylation, resulting in ATP depletion, making it impossible to produce enough energy to maintain basic cellular biochemical reactions (Zhao et al., 2022; Jiang et al., 2023). Our findings align with the literature mentioned earlier, indicating that ATP and MMP levels decrease when UCP2 expression is elevated. However, the decrease in ATP impedes the maintenance of normal cellular physiological functions, prompting mitochondria to accelerate ATP production in order to achieve normal cellular homeostasis. This leads to increased proton leakage during oxidative phosphorylation and elevated ROS levels, which in turn impairs mitochondrial function and causes oxidative damage to tissues. Therefore, targeting UCP2 therapeutically could serve as a strategy to disrupt the detrimental cycle of mitochondrial dysfunction and oxidative stress.

UCP2 serves as a vital controller of cellular fuel utilization and systemic glycolipid metabolism (Diano and Horvath, 2012). Multiple studies have highlighted the association between UCP2 overexpression and the initiation of metabolic disorders, emphasizing the crucial significance of managing UCP2 levels (Diano and Horvath, 2012). UCP2 typically participates in the modulation of oxidative phosphorylation within mitochondria, which affects intracellular energy metabolism. glucose transporter proteins is the major membrane protein for transporting glucose. GLUT4, extensively researched as the predominant glucose transport protein, exhibits abundant expression across various tissues and holds a critical function in maintaining glucose homeostasis. The research revealed that UCP2 hinders the progression of physiological retinal vascular development (PRVD) by disrupting glucose uptake through glucose transporter protein 1 (Han et al., 2019). Kutsche HS et al. found that silencing UCP2 improves glucose uptake and maintains cardiomyocyte function, improving heart failure (Kutsche et al., 2020). Inverse regulation between UCP2 and GLUT4 has been reported in several studies (Kato et al., 2004; Kutsche et al., 2020). Similar to previous studies, we found that GP inhibited the expression of UCP2, leading to increased levels of GLUT4 protein expression in cells and tissues, increased cellular glucose uptake, and alleviation of periodontal tissue damage.

There are few studies on the protective effect of GP on periodontal structure and function. GP has been shown to have beneficial antioxidant effects, including its ability to remove ROS and increase antioxidant enzyme activity (Li et al., 2020). In addition, GP has been shown to be a specific UCP2 inhibitor (Zhang et al., 2006). Studies have shown that GP can inhibit the production of MMP-1 and MMP-3 in TNF-α induced hPDLCs for the treatment of periodontal disease (Shindo et al., 2014). GP can also alleviate diabetic retinopathy by inhibiting UCP2 regulation of mitochondrial metabolism and glucose uptake (Sun et al., 2024). These results lay the groundwork for our investigation that GP can prevent periodontitis by inhibiting UCP2 to protect mitochondrial function and enhance glucose uptake. In this study, the therapeutic effect of GP was strongly linked to its ability to inhibit UCP2, thereby restoring mitochondrial function, promoting cellular glucose uptake, and reducing oxidative stress and periodontal tissue destruction.

In this study, we demonstrate that GP can alleviate oxidative damage of hPDLCs and periodontitis in rats. Potential mechanisms may involve the protective impact of GP on mitochondrial function and the augmentation of glucose uptake capacity, with UCP2 serving as a key player in this process. But in this study absence of UCP2 knockout experiments to confirm UCP2’s role in oxidative stress damage in hPDLCs, and the lack of further confirmation by using a gene defective animal model, is a limitation of our study. Secondly, from a clinical perspective, prioritizing local interventions for periodontal tissue to mitigate potential adverse effects on essential organs could present a safer and more effective treatment approach.

## 5 Conclusion

**FIGURE 7:**
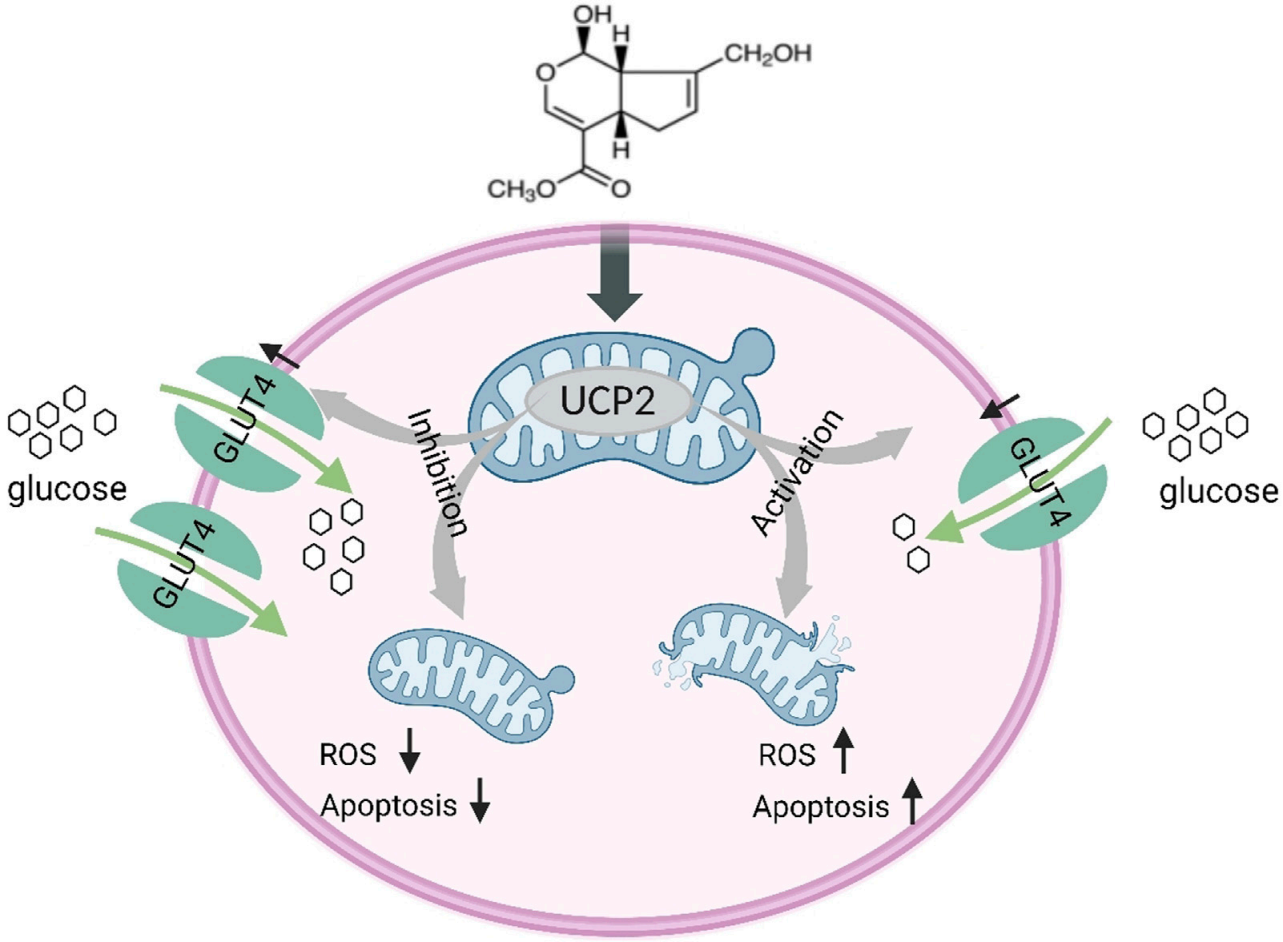
Schematic illustration of GP alleviating mitochondrial dysfunction and abnormal glucose uptake by inhibiting UCP2.

Our findings indicate that periodontitis elevates oxidative stress levels, initiates mitochondrial dysfunction and apoptosis, boosts UCP2 levels, and hampers the expression of glucose transporter proteins and glucose uptake. GP intervention effective in alleviating cell injury and periodontitis in rats. The potential mechanism may stem from GP’s protective influence on mitochondrial function and its facilitation of glucose transporter protein expression and glucose uptake, with UCP2 playing a pivotal role. Additionally, our discoveries offer robust support for preserving mitochondrial function and enhancing glucose uptake, potentially presenting a novel avenue for preventing periodontitis (Figure 7).

## Data Availability

The raw data supporting the conclusions of this article will be made available by the authors, without undue reservation.
