# Supplementary figures and images for: Genipin attenuates oxidative damage in periodontal tissues by alleviating mitochondrial dysfunction and abnormal glucose uptake through inhibition of UCP2
Source: Front Pharmacol. 2025 Mar 26;16:1446574. doi: 10.3389/fphar.2025.1446574 (PMC11979241; doi:10.3389/fphar.2025.1446574)

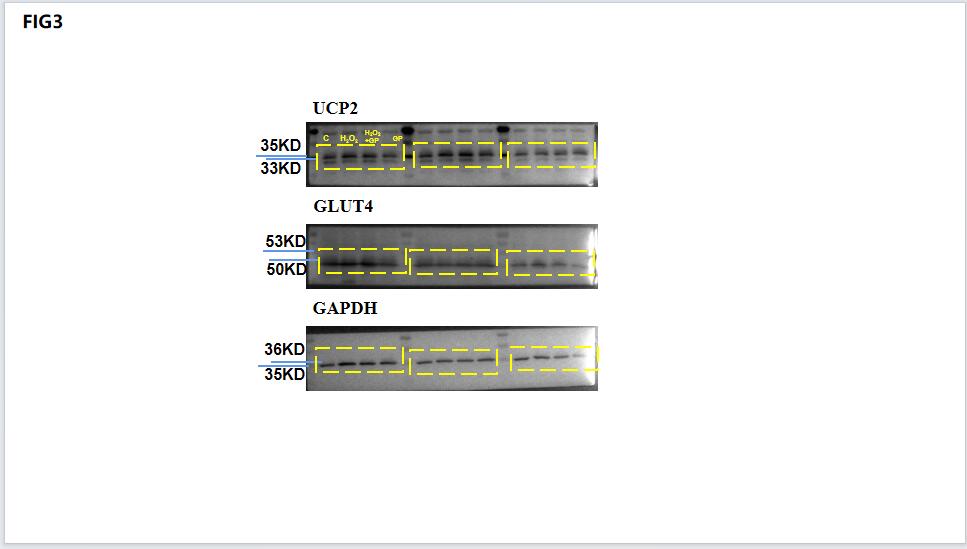

Supplement: Supplementary file 1 [file Image3.jpeg]

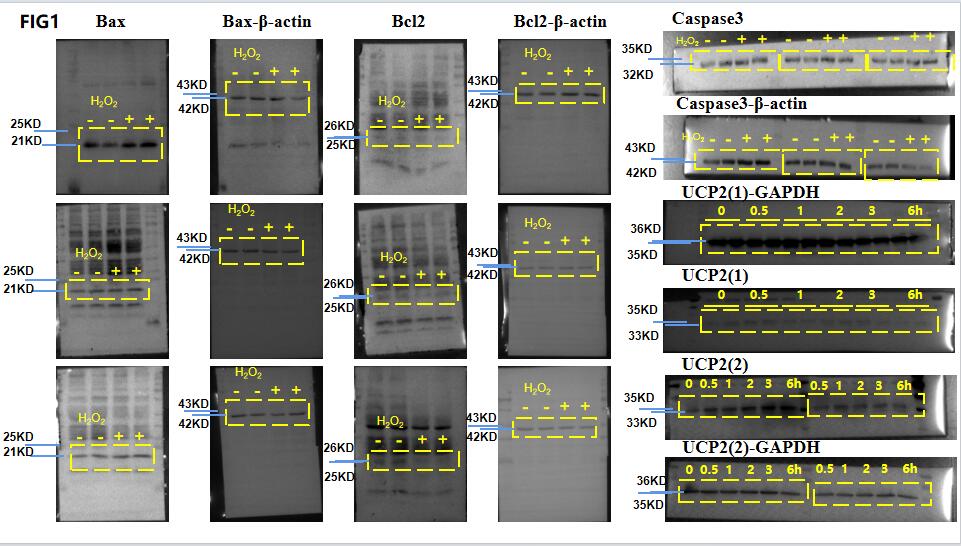

Supplement: Supplementary file 2 [file Image1.jpeg]

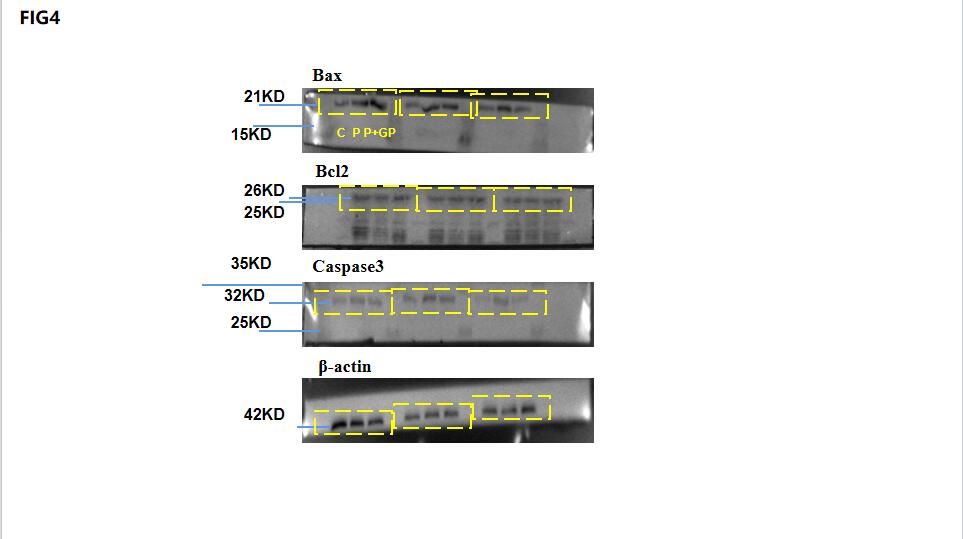

Supplement: Supplementary file 3 [file Image4.jpeg]

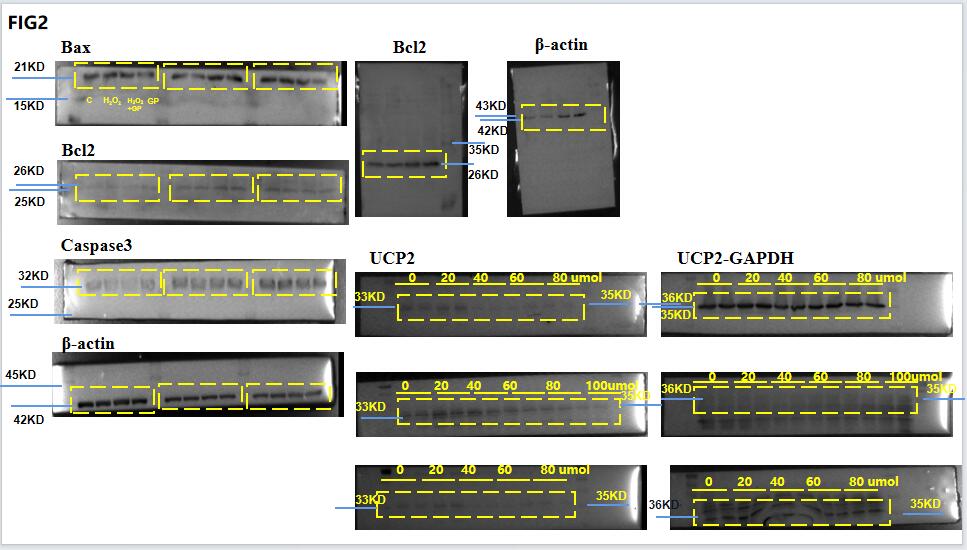

Supplement: Supplementary file 4 [file Image2.jpeg]

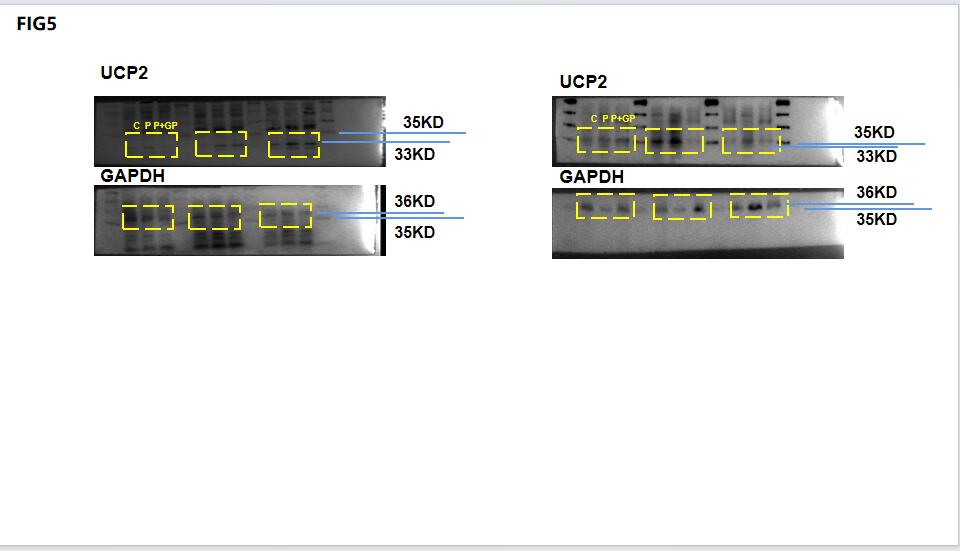

Supplement: Supplementary file 5 [file Image5.jpeg]

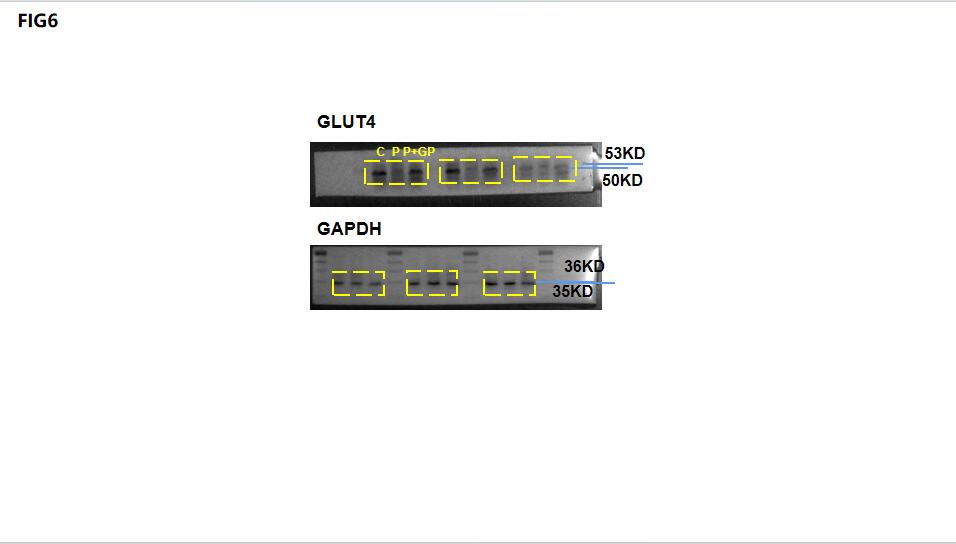

Supplement: Supplementary file 6 [file Image6.jpeg]
